# Genetic Polymorphisms Associated with Spontaneous Intracerebral Hemorrhage

**DOI:** 10.3390/ijms19123879

**Published:** 2018-12-04

**Authors:** Yi-Chun Chen, Kuo-Hsuan Chang, Chiung-Mei Chen

**Affiliations:** Department of Neurology, Chang Gung Memorial Hospital Linkou Medical Center and College of Medicine, Chang-Gung University, No. 5, Fuxing St., Guishan Township, Taoyuan County 333, Taiwan; asd108@adm.cgmh.org.tw (Y.-C.C.); gophy5128@cloud.cgmh.org.tw (K.-H.C.)

**Keywords:** intracerebral hemorrhage, genetics, association studies, ethnicities

## Abstract

Differences in the incidence of spontaneous intracerebral hemorrhage (ICH) between ethnicities exist, with an estimated 42% of the variance explained by ethnicity itself. Caucasians have a higher proportion of lobar ICH (LICH, 15.4% of all ICH) than do Asians (3.4%). Alterations in the causal factor exposure between countries justify part of the ethnic variance in ICH incidence. One third of ICH risk can be explained by genetic variation; therefore, genetic differences between populations can partly explain the difference in ICH incidence. In this paper, we review the current knowledge of genetic variants associated with ICH in multiple ethnicities. Candidate gene variants reportedly associated with ICH were involved in the potential pathways of hypertension, vessel wall integrity, lipid metabolism, endothelial dysfunction, inflammation, platelet function, and coagulopathy. Furthermore, variations in *APOE* (in multiple ethnicities), *PMF1/SLC25A44* (in European)*, ACE* (in Asian), *MTHFR* (in multiple ethnicities), *TRHDE* (in European), and *COL4A2* (in European) were the most convincingly associated with ICH. The majority of the associated genes provide small contributions to ICH risk, with few of them being replicated in multiple ethnicities.

## 1. Introduction

Spontaneous intracerebral hemorrhage (ICH) is a devastating stroke subtype, which accounts for 8–10% and 22–35% of all stroke patients in Western countries [1,2,3] and in the Asian population [4,5,6], respectively. An estimated 42% of the variance in ICH incidence was explained by ethnicity, given that ICH incidence in the Asian population (51.8 per 100,000 person-years) doubled that seen in Caucasians (24.2) [7]. The majority of ICH (65–80%) occurs in deep brain regions (DICH), including the basal ganglia, thalamus, brainstem, and cerebellum. Further, lobar ICH (LICH) and DICH differ based on both their risk factors and pathogenesis [8,9]. Risk factors for ICH vary by location of hemorrhage. Specifically, while LICH was partially attributable to cerebral amyloid angiopathy (CAA), particularly in the elderly, considering that the amyloid deposits are primarily located in cortical vessels, hypertension is the main cause of DICH instead (>80%) [10]. Other risk factors of ICH include excessive use of alcohol, which impairs coagulation and directly affects cerebral vessel integrity, low serum cholesterol levels, and other factors which cause coagulopathy or vasculopathy [8]. Interestingly, Caucasians reported a higher proportion of LICH (15.4% of all ICH) than did Asians (3.4%) [10]. Additionally, American blacks have higher ICH rates in young and middle-aged people than whites, particularly for DICH [11]. Further, men have younger age of ICH than women among Asians [12]. Differences in the exposure to causal factors, the control of hypertension and cholesterol, and the anticoagulant and antiplatelet treatment for stroke between countries and genders explain an important portion of the variance observed in ICH incidence between ethnicities [10] and genders [13,14,15,16]. However, a significant part of this variation remains unexplained. Approximately 30% of the risk factors of ICH were estimated to be undefined, or a result of a family history of such a condition [17]. ICH in a first-degree relative was reported to increase an individual’s odds for developing ICH six-fold. [18]. Additionally, monogenetic disorders associated with ICH account for less than 1% of all ICH and typically occur in childhood or young adults, such as genes related to cerebral cavernous malformations (CCMs) and cerebral autosomal dominant arteriopathy with subcortical infarcts and leukoencephalopathy (CADASIL) [19,20]. Given that one-third of the risk factors related to ICH can be explained by both common and rare genetic variations, the genetic differences between populations can partly elucidate the variation in ICH incidence observed [21]. Prior reports of ICH genetics are mainly approached by selecting candidate genetic variations, which are involved in the possible underlying pathogenic pathways and associated with potential environmental risk factors. However, many of the candidate genetic studies were limited by the small sample size, ethnicity disparities, and selection bias for phenotypes, leading to inconsistent results. Because of the advance in genotyping technologies, which are capable of assessing more than 500,000 to 1 million single nucleotide polymorphisms (SNPs) in a single sample, genome wide association studies (GWA) are now able to extensively examine the genetic factors of common diseases. The benefit of GWA is that it permits thousands of genome wide comparisons without a priori knowledge of target genetic function. Identification of genetic risk factors may shed light on possible underlying pathogenesis, potential therapeutic targets, and disease prevention strategies. Therefore, this review focuses on the available data reported in English on the PubMed, including candidate genetic studies (with number of ICH cases > 100), meta-analysis, and GWA to discuss the common genetic variations associated with ICH between ethnicities.

## 2. Common Genetic Predisposition of ICH

Candidate gene variants reported to be associated with ICH were potentially involved in the following pathways: hypertension, vessel wall integrity, lipid metabolism, endothelial dysfunction, inflammation markers, platelet function, and coagulopathy [22,23]. We will discuss the reported genetic variations according to the underlying mechanisms in the following sections. Genome Reference Consortium Human Build 38 (GRCh38) is used for denoting the genomic positions of the variations. 

### 2.1. Genetic Variants Related to the Renin–Angiotensin System (Table 1)

Hypertension represents the most critical risk factor of ICH, accounting for about 54% of ICH cases [18]. In fact, the renin–angiotensin system controls BP by regulating the volume of fluids in the body. Further, the angiotensin I converting enzyme (ACE) is an integral membrane protein which converts angiotensin I, a hormone, to angiotensin II, an active vasoconstrictor, leading to vasoconstriction and inhibition of bradykinin.

#### Angiotensin I Converting Enzyme (ACE)

Cytogenetic location: 17q23.3, 21 kb, main risk variant: rs1799752 (chr17:63488530–63488543, intron variant: Alu sequence). The rs1799752 variant implies either an insertion (I allele) or a deletion (D allele, lacks the repetitive element) of an Alu repetitive sequence in intron 16. When compared to the II genotype, the DD genotype correlates with increased ACE activity [24]. Additionally, increased vasoconstriction results in an increased risk of developing ICH. The largest meta-analysis [25] examined 33 studies (3355 ICH cases and 4722 controls), and reported the D allele to be a risk factor of ICH in the Asian population only (28 of 33 studies), as the same was not valid for the Caucasians [25,26]. In fact, Asian ICH patients were more likely to have hypertension than Caucasian patients [10]. Although we indicated the association between the D allele and DICH to be mediated by hypertension [27], the mechanisms that link the rs1799752 polymorphism to ICH, especially to LICH, are yet to be defined. Additionally, the D allele was described as the major allele in Caucasians [28], regardless of the absence of association between rs1799752 and the risk of developing ICH. Other mechanisms including inflammation [29], vascular remodeling [30], and the amyloid-β (Aβ) metabolism [31] may cause the disparities in *ACE* susceptibility found between ethnicities. 

**Table 1:** Variants associated with spontaneous intracerebral hemorrhage (ICH): hypertension and vascular integrity.

Gene Name and Abbreviation	Protein Function	Variant Locus	Population	No of Cases/Controls	MAF of Cases/Controls	OR (95% CI)	Ref	Notes
*Angiotensin-converting enzyme (ACE)*	Converts angiotensin I to angiotensin II	rs1799752: intron variant: Alu sequence	Asian	2941/3715	D 0.44/0.37	Rec: 1.98 (1.53–2.57); Dom: 1.31 (1.18–1.45)	[25]	Meta-analysis in LICH + DICH;
			Caucasian	414/1007	I 0.36/0.48 [28] I 0.57/0.47 [32]	No significance	[25,26]	Meta-analysis in LICH + DICH;
*Alpha-2 type IV collagen (COL4A2)*	Abundant component of the cerebral vasculature basement membranes.	intron variants: rs9521732 C>A; rs9521733 T>C; rs9515199 C>T	European	1545/1485	A 0.41/0.46; C 0.40/0.43; T0.41/0.47	Add: 1.28 (1.13–1.44); 1.29 (1.14–1.46); 1.28 (1.14–1.44)	[33]	Meta-analysis, Significant in DICH;
*Tissue metalloproteinase inhibitor 1 (TIMP-1)*	Inhibits matrix metalloproteinases and promotes cell proliferation.	rs2070584: intron variant A>C	Chinese	275/145 (male)	0.54/0.43	1.54 (1.03–2.3) (male)	[34]	LICH + DICH
		rs4898: intron variant T>C	Taiwanese	228/212 (male)	0.39/0.45	0.35 (0.15–0.81) (male)	[13]	DICH
*TIMP-2*		rs7503726: 5′ UTR variant G>A	German	45/253	0.4/0.37 (total stroke)	Rec: 2.02 (1.1–3.7)	[35]	LICH + DICH
		rs7503607: 5′ UTR variant C>A	Taiwanese	396/376	0.18/0.13	Add: 2.5 (1.37–4.38) (elder group)	[14]	DICH

MAF: Minor allele frequency (MAF), OR: odds ratio, Ref: references, Rec: recessive model, Dom: dominant model, Add: additive model, 5′ UTR: 5′ untranslated region.

### 2.2. Genetic Variants Related to Vessel Wall Integrity (Table 1)

With persistent hypertension, smooth muscle cell proliferation in the cerebral arterioles occurs with the formation of reactive hyperplasia, replaced by collagen tissues, on the vascular walls [30]. Specifically, when the deposition of collagen becomes insufficient, the arteriolar wall will dilate and result in a Charcot-Bouchard aneurysm, which weakens vascular integrity [36]. Bleeding of the aneurysm is mainly determined by the extent of the vascular pathological changes, individuals’ systemic blood pressure and other factors regulating hemostasis [8]. 

#### 2.2.1. Collagen, Type IV, Alpha-2 (COL4A2)

Cytogenetic location: 13q34, 206 kb, main risk variants: intron variants: rs9521732 (chr13:110381474, C>A), rs9521733 (chr13:110382195, T>C), and rs9515199 (chr13:110381357, C>T). Both *COL4A1* and *COL4A2* are highly expressed in the vascular basement membranes and were associated with an increased risk of small vessel diseases, especially ICH [33]. Specifically, three nonsynonymous coding mutations (COL4A2/ p.E1123G, p.Q1150K, and p.A1690T) were reported to contribute to the sporadic Caucasian cases of ICH in an autosomal dominant phenotype [37]. These mutations impaired both COL4A1 and COL4A2 secretion, and caused their intracellular retention within the endoplasmic reticulum (ER), leading to ER stress and cytotoxicity. Furthermore, other structurally-related variants (COL4A1/p.P352L and p.R538G) were described to trigger sporadic late-onset ICH [38]. 

A meta-analysis on the common genetic variation within both *COL4A1* and *COL4A2* among individuals of European ancestry identified three intronic SNPs (rs9521732, rs9521733, and rs9515199) in *COL4A2* to be significantly associated with DICH [33]. In contrast, there was no reported replication study in ethnic groups of non-European ancestry. 

#### 2.2.2. Tissue Inhibitors of Metalloproteinase-1 (TIMP-1) and TIMP-2

*TIMP-1* Cytogenetic location: Xp11.3, 4.5 kb, main risk variant: rs2070584 (chrX:47587120, intron variant, A>C) and main protective variant: rs4898 (chrX:47585586, intron variant, T>C). *TIMP-2* Cytogenetic location: 13q34, 206 kb, main risk variants (5′ untranslated region, 5′ UTR): rs7503726 (chr17:78925349, G>A) and rs7503607 (chr17:78925357, C>A).

Matrix metalloproteinases (MMPs) are a family of zinc/calcium-dependent endopeptidases which digest both the extracellular matrix (ECM) and the basal lamina [39], causing a blood-brain barrier breakdown and activating an inflammatory reaction [40]. Among MMPs, gelatin-binding MMPs were particularly unique in BBB damage because of their ability to digest type IV and type V collagen, which are the essential constituents of vascular basement membrane in the vascular endothelium. Degradation of the collagen tissues may break down the vessel integrity, which is responsible for the eventual rupture of the vessel walls [41,42,43]. MMP activity is suppressed by tissue inhibitors of metalloproteinases (TIMPs). Each TIMP was able to interact with any of the MMPs; however, certain combinations between MMPs and TIMPs have been reported, in which TIMP-1 is the main endogenous inhibitor to MMP-9 while TIMP-2 is to MMP-2 [43,44]. A mouse model showed neuroprotection by inhibition of MMPs in acute ICH, suggesting a therapeutic strategy for the treatment of acute brain injury after ICH [45]. Additionally, TIMP-1 and TIMP-2 were shown to have a protective role for the progression of cerebral aneurysms, which suggests that TIMPs may help prevent the degradation of ECM and rupture of cerebral aneurysms [46]. Considering that *TIMP-1* locates to the X chromosome, its effect is more prominent in males than females. Further, while an intronic polymorphism of *TIMP-1* rs2070584 C allele is associated with ICH in Chinese male patients [34], C allele of rs4898 provides a protective effect on DICH risk in the elderly male group [13]. Additionally, although the *TIMP-2* AA rs7503726 genotype increased the risk of developing ICH in a genetic recessive model conducted on the German population [35], it was considered a protective factor against DICH in elderly Taiwanese females [14]. The disparity between the two reports may be due to different minor allele frequency (MAF) (39.14% in the former and 49.7% in the latter), heterogeneity of phenotypes, and stratification of subgroups in the latter report. Overall, the imbalance between MMPs and TIMPs may be responsible for ECM degradation, resulting in the progression and rupture of damaged vessels. 

### 2.3. Genetic Variants Related to Lipid Metabolism (Table 2)

#### 2.3.1. Apolipoprotein E (APOE)

Cytogenetic location: 19q13.32, 3 kb, main risk variant: ε2 and ε4. *APOE* ε2/ε3/ε4 alleles are haplotypes constructed by two missense variants, rs7412 (chr19:44908822, C>T) and rs429358 (chr19:44908684, T>C). *APOE* ε3ε3 is the most common genotypes. Both ε2 and ε4 are missense variants. The *APOE* gene produces three apolipoprotein (APOE) isoforms which interact differently with specific lipoprotein receptor and thus influence cholesterol level. In general, the ε2 allele is associated with lower serum total cholesterol levels, and ε4 raises them [47]. APOE transports lipoproteins, fat-soluble vitamins, and cholesterol, and is involved in cell membrane maintenance and repair [48]. The ε2 or ε4 alleles are associated with an increase in both β-amyloid protein deposition and fibrinoid necrosis in the vessel wall, which augments the vasculopathy effects of amyloid deposition in cerebral vessels [49]. A population study suggested that carrying either the ε2 or the ε4 raised the risk of developing both DICH and LICH [50]. Specifically, Asian carriers had a doubled risk of developing DICH, whereas Europeans reported a tripled risk of LICH [50]. Additionally, a genome-wide association study conducted on the European population (2189 ICH cases and 4041 controls) identified both the ε2 and the ε4 to be risk factors for developing LICH, whereas ε4 was associated with DICH [51]. Furthermore, another meta-analysis including 11 case–control studies (1238 ICH cases and 3575 controls) showed that ICH cases had a significantly higher frequency of the *APOE* ε4 allele [52]. Specifically, ICH cases had a significantly higher frequency of the *APOE* ε4 allele in both Asians and Caucasians. However, they did not find a significant relationship between the *APOE* ε2 allele and the risk of ICH [52]. A meta-analysis of 58 studies (6855 participants), investigating both the *APOE* genotype and sporadic CAA, showed convincing evidence of a dose-dependent association between the ε4 and sporadic CAA [53], consistent with their associations with LICH. 

#### 2.3.2. ER Lipid Raft Associated 1 (ERLIN1)

Cytogenetic location: 10q24.31, 35 kb, main protective variant (upstream variant): rs1324694 (chr10:100186688, C>T). ERLIN1 is a component of lipid rafts, and is specifically localized to the endoplasmic reticulum and the nuclear envelope. ERLIN1 is involved in cellular cholesterol homeostasis and in defining the lipid-raft-like domains of the endoplasmic reticulum [54]. The association between rs1324694 and ICH was found in a Japanese cohort [54], in which the rs1324694 minor allele (T) in the 5′ region of *ERLIN1* was significantly related to ICH, and was reported to have a protective role. However, to date, a replication study has not yet been reported.

#### 2.3.3. Low-Density Lipoprotein Receptor (LDLR)

Cytogenetic location: 19p13.2, 44 kb, main protective variant (synonymous variant): rs688 (chr19:11116926, C>T). According to the Ensemble annotation, the most severe consequence of rs688 is a synonymous variant. The LDLR is a cell surface receptor that plays an important role in cholesterol homeostasis. The minor rs688 variant is an intron variant that directly affects exon 12 alternative splicing, and is related to increases in plasma cholesterol levels [55]. Its association with ICH was found in a single Taiwanese cohort, which suggested the homozygous minor allele T to be correlated with a 73% decreased risk of developing ICH [56]. 

#### 2.3.4. Apolipoprotein (a) (LPA)

Cytogenetic location: 6q25-q26, 134 kb, main risk pentanucleotide variant: TTTTA repeat in 5′ UTR −1373 upstream of the transcription start site. *LPA* encodes apolipoprotein (a) which forms the lipoprotein Lp(a), together with an LDL-like lipid core. Lp(a) level is mainly controlled by *LPA* expression, which is associated with atherosclerosis and inhibition of thrombolysis. Additionally, Lp(a) is a structural analogue of plasminogen and can compete with it for fibrin binding, thereby suppressing fibrinolysis. Although high Lp(a) levels were associated with lower risk of major bleeding in the brain in the Copenhagen General Population study [57], a low number of TTTTA repeats was shown to be related to an elevation in the levels of Lp(a) and to an increased risk of ICH in the Chinese population [58]. The contradictory results require more studies in different ethnic groups, involving larger cohorts, to clarify further.

**Table 2:** Variants associated with ICH: lipid metabolism.

Gene Name and Abbreviation	Protein Function	Variant Locus	Population	No of Cases/Controls	MAF of Cases/Controls	OR (95% CI)	Ref	Notes
*Apolipoprotein E (APOE)*	Involved in lipid transport and metabolism, and cell membrane maintenance and repair.	Haplotypes constructed by rs7412 and rs429358. APOE ε2: missense variant	Caucasian	2189 ICH cases and 4041 controls	0.09–0.15/0.07–0.1	LICH: 1.82 (1.50–2.23)	[51]	GWA in LICH and DICH
		APOE ε4: missense variant	Caucasian	2189 ICH cases and 4041 controls	0.12–0.24/0.08–0.19	LICH: 2.20 (1.85–2.63); DICH: 1.21 (1.08–1.36)	[51]	GWA in LICH and DICH
			Caucasian	539/1573	0.22/0.17 carrier frequency	1.34 (1.07, 1.66)	[52]	Meta-analysis, LICH + DICH
			Asian	699/2002	0.11/0.09 carrier frequency	1.52 (1.20, 1.93)	[52]	Meta-analysis, LICH + DICH
*ER lipid raft associated 1 (ERLIN1)*	Components of lipid rafts localized to the endoplasmic reticulum and nuclear envelope	rs1324694: upstream variant C>T	Japanese	373/3665	6.4/9.9	Dom: 0.59 (0.39–0.88)	[54]	LICH + DICH
*Low-density lipoprotein receptor (LDLR)*	Cholesterol hemostasis	rs688: synonymous variant C>T	Taiwanese	447/430	0.18/0.18	Rec: 0.27 (0.10–0.79)	[56]	LICH + DICH
*Apolipoprotein(a) (LPA)*	Atherogenicity, Inhibits tissue type plasminogen activator-1	TTTTA repeat in 5′ UTR	Chinese	499/1817	-	1.62 (1.09–2.37)	[58]	LICH + DICH

MAF: Minor allele frequency (MAF), OR: odds ratio, Ref: references, Rec: recessive model, Dom: dominant model, Add: additive model, GWA: Genome-wide association study, 5′ UTR: 5′untranslated region.

### 2.4. Genetic Variants Related to Inflammation (Table 3)

Endothelium has functions in the regulation of vascular tone and inflammatory balance. The loss of endothelial-mediated vasodilatation, and the presence of both the inflammatory and prothrombotic states, are the earliest manifestations of vascular damage.

#### 2.4.1. Methylenetetrahydrofolate Reductase (MTHFR)

Cytogenetic location: 1p36.22, 20 kb, main risk variant: rs1801133 (chr1:11796321, missense, C>T, p.A222V). The MTHFR plays a role in processing amino acids and converting their homocysteine to methionine. The rs1801133 polymorphism in the *MTHFR* exon 4 reduces the MTHFR activity and leads to hyperhomocysteinemia [59], a risk factor for atherosclerosis, inflammation, and endothelial dysfunction [60]. The largest of the meta-analyses (16 studies on 1585 cases/3620 controls (Asians) and 243 cases/447 controls (Caucasians)) [61] reported an association between the *MTHFR* 677 T variant allele and ICH in both the Asian (in all the inheritance models) and the Caucasian (in the recessive model) populations, with a stronger correlation in the Asian than in the European population. 

#### 2.4.2. Interleukin 6 (IL6)

Cytogenetic location: 7p15.3, 6 kb, main risk variant (intron variant, upstream variant): rs1800796 (chr7:22726627, G>C). IL-6 is a pleiotropic cytokine which may be a key mediator in the inflammatory response to ICH [62]. It in fact both activates endothelial cells, and induces vascular dysfunction, vascular macrophage accumulation, oxidative stress and increased angiotensin I receptors in vascular smooth muscle. This, in turn, enhances NF-κB activation [63], which leads to increased expression of pro-inflammatory cytokines. The functional promoter of *IL-6* in the rs1800796 polymorphism (C allele) was significantly associated with a higher risk of developing ICH in the Japanese population [64]. It is noteworthy that the C allele is the major allele of rs1800796 in the Asian population (79%) but the minor allele in the European population (< 5%), according to the HapMap database. While this genetic variant may be related to an increased expression of *IL-6* [65], a high plasma IL-6 level at hospital admission was considered as an independent predictor of hematoma enlargement [66].

#### 2.4.3. Tumor Necrosis Factor (TNF)

Cytogenetic location: 6p21.33, 6 kb, main variants: rs1799964 (chr6:31574531, T>C) (−1031); rs1800629 (chr6:31575254, G>A) (−308); rs1800630 (chr6:31574699, C>A) (−863). TNF-α is one of the main proinflammatory cytokines, and plays a central role in initiating and regulating the inflammatory response. Polymorphisms in the regulatory region result in different TNF-α concentrations. The above three SNPs were shown to be associated with increasing *TNF-α* expression [67,68]. TNF-α induces MMP production, leading to endothelial dysfunction and blood–brain barrier breakdown [69]. While two polymorphisms within the *TNF-α* promoter, namely rs1799964 and rs1800629, were associated with DICH in men, the risk of developing DICH was inversely associated with the rs1800630 polymorphism in the Taiwanese population [70]. In contrast, −857C/T (rs1799724) but not −308G/A was shown to be involved in male ICH susceptibility in a small Korean study [71].

#### 2.4.4. Trafficking Protein Particle Complex 9 (TRAPPC)

Cytogenetic location: 6p21.33, 2 kb, main protective variant: rs12679196 (chr8:139800104, intron variant, C>T). The protein encoded by *TRAPPC* is implicated in vesicular transport, and plays a role in neuronal NF-κB signaling by both binding the mitogen-activated protein kinase and inhibiting the κ light polypeptide gene enhancer in B-cells, i.e., kinase β [72]. Overexpression of *TRAPPC* potentiates TNF-α-induced NF-κB activation, through increased phosphorylation of the IKK complex and its downstream IκBα and p65 substrates [72]. *TRAPPC* encoded protein is therefore suggested to be an enhancer of the cytokine-induced NF-κB signaling pathway. In the Japanese population, an intronic polymorphism, namely rs12679196 (C allele), is significantly associated with ICH, while the T allele protects against the disease [54]. 

#### 2.4.5. Endoglin (ENG)

Cytogenetic location: 9q34.11, 39 kb, main risk variant: GGGGGA insertion. The *ENG* gene encodes a transmembrane glycoprotein, essential for angiogenesis and vascular development, which is predominantly expressed in vascular endothelial cells and is crucial for maintaining vascular integrity [73]. It functions as a co-receptor for transforming growth factor-β (TGFB) family members, and it interacts with their signaling serine/threonine kinase receptors [74]. Mutations in *ENG* were shown to represent a genetic marker of angiogenesis in hereditary hemorrhagic telangiectasia type 1 [75]. Soluble ENG is related to the formation of sporadic brain arteriovenous malformations by acting as a decoy receptor, which results in TGFB signaling inhibition [76]. A study conducted in the United States of America found a homozygous insertion of GGGGGA located 26 bases beyond the 3′ end of exon 7 of *ENG* to be associated with ICH [77]. 

#### 2.4.6. Interferon Epsilon (IFNE)

Cytogenetic location: 9p21.3, 1 kb, main risk variant: rs2039381 (chr9:21481484, C>T, stop gained, p.Q71Stop). Type I Interferons have major roles in the innate immune responses. In stroke, type I interferons, including IFNE, have a role in the cytotoxic immune pathway to control immune responses in the central nervous system [78]. *IFNE* is expressed in many tissues, including the brain, coronary smooth muscle endothelial cells, and microvascular endothelial cells [79]. It can be induced by proinflammatory cytokines and regulate the hyaluronic acid-mediated motility receptor which is involved in the formation of cerebral microvessels [80]. In the Korean population, a nonsense polymorphism of *IFNE*, rs2039381, is associated with ICH [81]. 

#### 2.4.7. Transforming Growth Factor Beta 2 Receptor 2 (TGFBR2)

Cytogenetic location: 3p24.1, 87 kb, main risk variant: rs2228048 (chr3:30672350, synonymous codon, C>T, N389N). TGFB is a cytokine that plays important roles in the development, homeostasis, and tolerance of T cells [82]. The *TGFBR2* is mainly expressed by neurons [83], and a reduction in its signaling results in accelerated age-dependent neurodegeneration [84]. A previous study in the Korean population found an association between the rs2228048 polymorphism and ICH [85]. 

**Table 3:** Variants associated with ICH: inflammation.

Gene Name and Abbreviation	Protein Function	Variant Locus	Population	No of Cases/Controls	MAF of Cases/Controls	OR (95% CI)	Ref	Notes
*Methylenetetrahydrofolate reductase (MTHFR)*	Converts homocysteine to methionine	rs1801133 C>T, p.A222V	Asian	1585/3620	0.48/0.41	1.42 (1.19–1.69)	[61]	Meta-analysis in LICH + DICH;
			Caucasian	243/447	0.18/0.48	Rec: 2.23 (1.06–4.71)	[61]	
*IL-6 (IL6)*	Proinflammatory cytokine	rs1800796: intron variant, G>C (−572)	Japanese	282/2010	0.19/0.25	Rec: 1.6 (1.2–2.1)	[64]	LICH + DICH
*Tumor necrosis factor (TNF)*	Proinflammatory cytokine; regulator of cell proliferation, lipid metabolism, apoptosis, and coagulation	rs1799964: downstream variant 500B, upstream variant 2KB T>C (−1031) rs1800629: upstream variant G>A (−308) rs1800630: downstream variant 500B, upstream variant 2KB C>A (−863)	Taiwanese	177/226 (male); 177/226 (male); 83/142 (female)	0.19/0.13 (male); 0.15/0.09 (male); 0.18/0.23 (female)	Add: 1.9 (1.1–3.4); 2.6 (1.3–5.3); 0.5 (0.2–0.9)	[70]	DICH
*Trafficking protein particle complex 9 gene (TRAPPC)*	Trafficking protein particle complex subunit 9	rs12679196: intron variant C>T	Japanese	376/3722	0.18/0.21	Add: 0.2 (0.0–0.6)	[54]	LICH + DICH
*Endoglin (ENG)*	Transmembrane glycoprotein, part of TGF-β receptor complex	GGGGGA insertion	US	103/202	0.09/0.02 (homozygous)	4.8 (1.3–21.6)	[77]	LICH + DICH
*Interferon epsilon (IFNE)*	Proinflammatory cytokines	rs2039381, stop gained C>T, p.Q71Stop	Korean	145/401	0.22/0.15	Add: 2.0 (1.3–3)	[81]	LICH + DICH
*Transforming growth factor beta 2 receptor 2 (TGFBR2)*	Transmembrane protein for development of T cells and regulator of cell proliferation	rs2228048: synonymous codon C>T, N389N	Korean	127/395	0.28/0.19	1.7 (1.2–2.4)	[85]	LICH + DICH

MAF: Minor allele frequency (MAF), OR: odds ratio, Ref: references, Rec: recessive model, Dom: dominant model, Add: additive model.

### 2.5. Other Genetic Variants (Table 4)

A meta-analysis of data from the International Stroke Genetics Consortium (1681 cases, 2261 controls) identified 1q22 to be a susceptibility locus for DICH [86]. Specifically, two genes are found at this locus, the *polyamine-modulated factor 1* (*PMF1*) and the *solute carrier family 25-member 44* (*SLC25A44*). The top-associated variant within this locus was rs2984613, an intron variant of *PMF1.* In the discovery phase of the meta-analysis, 12q21.1 was associated with ICH, especially with LICH, while the greatest association with rs11179580 was seen near the *thyrotropin-releasing hormone degrading enzyme* gene (*TRHDE*). However, the latter relation was not found to be significant in the replication phase.

#### 2.5.1. Polyamine-Modulated Factor 1 (PMF1)

Cytogenetic location: 1q22, 27 kb, main risk variant: rs2984613 (chr1:156227589, intron variant, C>T). *Solute carrier family 25, member 44 (SLC25A44)*: Cytogenetic location: 1q22, 18 kb. While the PMF1 protein, a nuclear protein regulated by polyamines, is involved in chromosomal alignment and segregation during mitosis [87], it also mediates the transcriptional induction of an acetyltransferase responsible for the rate-limiting enzyme in the catabolic pathway of polyamine metabolism, which has been implicated in breakdown of the blood–brain barrier and regulation of the excite-toxicity after stroke [88,89]. *SLC25A44* codes for solute carrier family 25 member 44 that belongs to the SLC25 family of mitochondrial carrier proteins, and is widely expressed in the central nervous system [90]. However, the pathological link between this protein and ICH is unknown to date. 

#### 2.5.2. Thyrotropin-Releasing Hormone Degrading Enzyme Gene (TRHDE)

Cytogenetic location: 12q21.1, 579 kb, main risk variant: rs11179580 (chr12:73192799, intron variant, C>T). The thyrotropin-releasing hormone (TRH) is a central neurotransmitter that stimulates hormone secretion from adenohypophyseal cells and is inactivated instead by the TRH-degrading enzyme [91]. The association between rs11179580 and ICH was discovered in the discovery phase of meta-analysis of six genome-wide association studies in Europeans; however, the result should be explored further given lack of replication confirmation [86].

#### 2.5.3. Fibrinogen Alpha Chain (FGA)

Cytogenetic location: 4q31.3, 7 kb, main risk variant: rs6050 (chr4:154586438, missense, T>C, NP_000499.1: p.T331A). Soluble fibrinogen is converted into insoluble fibrin by activated thrombin. The rs6050 polymorphism of *FGA* causes f substitution from threonine to alanine and alters the fibrinogen structure, leading to a reduced affinity for degrading enzymes, which increases, in turn, fibrin clot resistance to thrombolytic cleavage [92]. The rs6050 polymorphism was significantly associated with decreased plasma fibrinogen and reduced platelet distribution in white people [93]. This procoagulant *FGA* polymorphism was found to represent a risk factor of ICH in a Polish and Greek study [94]. Furthermore, haplotypes composed of the ATA (rs1800790 + rs1800787 + rs6050), AA (rs1800790 + rs6050), and TA (rs1800787 + rs6050), however not individual polymorphisms, were shown to contribute to the risk of ICH in the Chinese population as well [95].

#### 2.5.4. Tubulin Beta-1 Chain (TUBB1)

Cytogenetic location: 20q13.32, 10 kb, main risk variant: rs415064 (chr20:59022916, missense, G>C, p.Q43P). The TUBB1 is a microtubules component required for optimal platelet assembly and mutations in this gene could cause macrothrombocytopenia [96]. The rs415064 polymorphism in the *TUBB1* alters platelet reactivity by modulating platelet function and structure [97]. A study conducted on the Spanish population reported that the rs415064 polymorphism increases the risk of ICH and is associated with an earlier age at onset of ICH [98]. Furthermore, when the rs415064 polymorphism is inherited along with the −323 I/D polymorphism on the factor VII gene, the ICH risk increases 20-fold [98]. 

#### 2.5.5. WNK Lysine Deficient Protein Kinase 2 (WNK2)

Cytogenetic location: 9q22.31, 143 kb, main risk variant: rs16936752 (chr9:93301408, intron variant, T>G). The *WNK2* encodes a regulator of cell cycle progression. Wnk2 is a serine-threonine kinase which phosphorylates the exogenous substrate of the myelin basic protein, mostly on the serine residues. WNK2 is also involved in the modulation of growth factor-induced cancer cell proliferation [99]. However, no known function is associated with the pathogenesis of ICH to date. The rs16936752 polymorphism T allele of *WNK2* is an intron variant which was reported to increase the risk of ICH in the Japanese population [54]. 

#### 2.5.6. Potassium Channel, Subfamily K, Member 17 (KCNK17)

Cytogenetic location: 6p21.2, 15 kb, main protective variant (intron variants): rs12214600 (chr6:39300960, C>T) and rs10947803 (merged into rs9471058, chr6:39302834, C>A). KCNK17 is a member of the 2-pore domain superfamily of background potassium channels. It generates the negative membrane potential, contributes to the resting potential in excitable and non-excitable cells, and may influence cerebral blood vessel dilation. The T carrier of rs12214600 is associated with reduced risk of ICH, whereas the A carrier of rs10947803 increases the risk of ICH in the Chinese population [100,101]. 

**Table 4:** Variants associated with ICH: others.

Gene Name and Abbreviation	Protein Function	Variant Locus	Population	No of Cases/Controls	MAF of Cases/Controls	OR (95% CI)	Ref	Notes
*Polyamine-modulated factor 1 (PMF1)*	Required for chromosome alignment and segregation, and kinetochore formation during mitosis	rs2984613: intron variant C>T	European	1545/1481 664 LICH and 881 DICH cases	0.31/0.31	Add: 1.29 (1.22–1.46)	[86]	DICH; Meta-analysis of GWAs with replication
*Solute carrier family 25, member 44 (SLC25A44)*	Nuclear-encoded transporters embedded in the inner mitochondrial membrane and other organelle membranes	Within the susceptibility locus 1q22						
*Thyrotropin- releasing hormone- degrading ectoenzyme (TRHDE)*	Inactivates thyrotropin-releasing hormone	rs11179580: intron variant C>T	European	1545/1481 664 LICH and 881 DICH cases	0.24/0.25	Add: LICH: 1.56 (1.33–1.84); DICH: 1.25	[86]	LICH>DICH; Meta-analysis of GWAs without replication
*Fibrinogen alpha chain (FGA)*	Cleaved to yield monomers, which, together with fibrinogen beta and gamma, polymerize to form fibrin matrix	rs6050: missense T>C, p.T331A	Polish and Greek	503/774	0.21/0.23	Dom: 2.3 (1.1–4.8)	[94]	LICH + DICH
*Tubulin beta-1 chain (TUBB1)*	Major constituent of microtubules	rs415064: missense G>C, p.Q43P	Spanish	259/449	0.12/0.06	2.36 (1.25–4.45)	[98]	LICH + DICH
*WNK lysine deficient protein kinase 2 (WNK2)*	Serine/threonine kinase that controls PAK1, a regulator of cell motility	rs16936752: intron variant T>G	Japanese	376/3671	0.08/0.11	Rec: 1.59 ^#^ (1.10–2.38)	[54]	LICH + DICH
*KCNK17*	potassium channel, subfamily K, member17	rs12214600: intron variant C>T	Chinese	182/174	0.10/0.17	0.56 (0.35–0.90)	[100]	LICH + DICH
		rs10947803: (merged into rs9471058) intron variant C>A	Chinese	166/156	0.42/0.34	Dom: 1.65 (1.04–2.62)	[101]	LICH + DICH

MAF: Minor allele frequency (MAF), OR: odds ratio, Ref: references, Rec: recessive model, Dom: dominant model, Add: additive model, GWA: Genome-wide association study. ^#^ The risk allele is T.

## 3. Clinical Implications for ICH Management

Pharmacogenomics and pharmacogenetics enable clinicians and researchers to implement knowledge in the context of personalized medicine. Knowing gene–drug–disease relationships helps move treatment of disease from bench to bedside [102]. Although there was no report directly addressing pharmacogenomics/pharmacogenetics for ICH, several reports showed genetic variations related to the ICH risks or the treatments involving ICH prevention. Among the reviewed genetic variations, the *ACE* rs1799752 minor allele displayed significantly different response to captopril in type 2 diabetes mellitus, heart failure, and chronic obstructive pulmonary disease [102,103]. For another example, *APOE* rs7412 showed that heterozygous allele CT of rs7412 has a 39.9% lowering of LDL by atorvastatin, compared to a 36.4% lowering among Caucasians with the common allele CC [104]. Although *MTHFR* rs1801133 has an impact on clinical drug responses, the effects are mainly on cyclophosphamide and carboplatin in treating cancers [105,106]. Additionally, the *TNF* rs1800629 variant demonstrated different drug responses to TNF-alpha inhibitors in arthritis, psoriatic arthritis, and ankylosing spondylitis [107,108]. Whether polymorphisms are associated with different responses to treatments in ICH remains to be explored. 

## 4. Conclusions

The majority of the genes associated with ICH only provide small contributions to the risk of developing such a condition. However, the mechanisms behind such associations require further investigation. Additionally, variations in *APOE* (in multiple ethnicities), *PMF1/SLC25A44* (in Europeans), *ACE* (in Asians), *MTHFR* (in multiple ethnicities), *TRHDE* (in Europeans), and *COL4A2* (in Europeans) are the most convincing genetic factors related to ICH. Furthermore, although LICH and DICH differ in pathogenesis, many of the above-mentioned studies did not separate the ICH phenotypes. Except for *TIMP-1*, which locates to the X chromosome with effects more prominent in males than females, no report with adequate sample number has reported significant gender difference in genetic difference regarding ICH risks. Future replications are required to both confirm their association in multiple ethnicities and to elucidate the roles of these genetic variations in ICH pathogenesis. More importantly, given that both the lifestyle and the medication controls are different among countries, gene–gene and gene–environment interactions should be taken into consideration in the analyses.
